# Standardised packs and larger health warnings: visual attention and perceptions among Colombian smokers and non‐smokers

**DOI:** 10.1111/add.15779

**Published:** 2022-01-08

**Authors:** Carlos Sillero‐Rejon, Osama Mahmoud, Ricardo M. Tamayo, Alvaro Arturo Clavijo‐Alvarez, Sally Adams, Olivia M. Maynard

**Affiliations:** ^1^ School of Psychological Science University of Bristol Bristol United Kingdom; ^2^ Health Economics Bristol, Population Health Sciences, Bristol Medical School University of Bristol Bristol United Kingdom; ^3^ National Institute for Health Research Applied Research Collaboration West (NIHR ARC West) University Hospitals Bristol NHS Foundation Trust Bristol United Kingdom; ^4^ Department of Mathematical Sciences University of Essex Colchester United Kingdom; ^5^ Department of Applied Statistics Helwan University Egypt; ^6^ Departamento de Psicologia Universidad Nacional de Colombia Bogotá Colombia; ^7^ Department of Psychology University of Bath Bath UK; ^8^ MRC Integrative Epidemiology Unit University of Bristol Bristol United Kingdom

**Keywords:** Colombia, discrete choice, eye‐tracking, health warnings, plain packaging, South America, standardised packaging, tobacco

## Abstract

**Aims:**

To measure how cigarette packaging (standardised packaging and branded packaging) and health warning size affect visual attention and pack preferences among Colombian smokers and non‐smokers.

**Design:**

To explore visual attention, we used an eye‐tracking experiment where non‐smokers, weekly smokers and daily smokers were shown cigarette packs varying in warning size (30%‐pictorial on top of the text, 30%‐pictorial and text side‐by‐side, 50%, 70%) and packaging (standardised packaging, branded packaging). We used a discrete choice experiment (DCE) to examine the impact of warning size, packaging and brand name on preferences to try, taste perceptions and perceptions of harm.

**Setting:**

Eye‐tracking laboratory, Universidad Nacional de Colombia, Bogotá, Colombia.

**Participants:**

Participants (*n* = 175) were 18 to 40 years old.

**Measurements:**

For the eye‐tracking experiment, our primary outcome measure was the number of fixations toward the health warning compared with the branding. For the DCE, outcome measures were preferences to try, taste perceptions and harm perceptions.

**Findings:**

We observed greater visual attention to warning labels on standardised versus branded packages (*F*[3,167] = 22.87, *P* < 0.001) and when warnings were larger (*F*[9,161] = 147.17, *P* < 0.001); as warning size increased, the difference in visual attention to warnings between standardised and branded packaging decreased (*F*[9,161] = 4.44, *P* < 0.001). Non‐smokers visually attended toward the warnings more than smokers, but as warning size increased these differences decreased (*F*[6,334] = 2.92, *P* = 0.009). For the DCE, conditional trials showed that increasing the warning size from 30% to 70% reduced preferences to try (odds ratio [OR] = 0.48, 95% CI = [0.42,0.54], *P* < 0.001), taste perceptions (OR = 0.61, 95% CI = [0.54,0.68], *P* < 0.001); and increased harm perceptions (OR = 0.78, 95% CI = [0.76,0.80], *P* < 0.001). Compared with branded packaging, standardised packaging reduced our DCE outcome measures with ORs ranging from OR = 0.25 (95% CI = [0.17,0.38], *P* < 0.001) to OR = 0.79 (95% CI = [0.67,0.93], *P* < 0.001) across two brands. These effects were more pronounced among non‐smokers, males and younger participants. Unconditional trials showed similar results.

**Conclusions:**

Standardised cigarette packaging and larger health warnings appear to decrease positive pack perceptions and have the potential to reduce the demand for cigarette products in Colombia.

## INTRODUCTION

Smoking causes 10% of all deaths among Colombian adults and the prevalence of smoking among children is higher than in other middle‐income countries [1, 2]. Smoking‐related morbidity and mortality are projected to increase in Colombia over the next decade [3, 4]. To warn adults and children about the risks of smoking, the World Health Organisation (WHO) recommends that pictorial warnings cover at least 50% of the pack and that standardised packaging replaces branded packaging [5]. In Colombia, standardised packaging has yet to be introduced and pictorial warnings (which have featured on packs since 2010 and are changed annually) cover only 30% of the pack surface, thereby only complying with the WHO minimum requirements [5]. In countries where these measures have been implemented, there is evidence that they (i) make smoking less appealing, especially among young people [6, 7, 8], (ii) increase warning noticeability [7, 9, 10], (iii) prevent people from being misled about the health risks of smoking [7, 10, 11] and (iv) ultimately lead to smoking cessation [8, 12, 13].

Health warnings and standardised packaging influence smoking behaviour by changing smoking intentions, attitudes and knowledge about the health risks. Before this, however, warnings must first be noticed and must provoke a series of reactions (e.g., negative affect) [10, 12]. Visual attention to warnings is, therefore, a critical first step in warning engagement [10, 14, 15]. Eye‐tracking is considered a gold standard objective measure of attention to tobacco warnings [14, 16, 17, 18]. Our previous research found that whereas daily smokers avert their gaze from warnings (i.e., avoid warnings), standardised tobacco packaging increases visual attention to health warnings among non‐smokers and non‐daily smokers [9, 19, 20]. Non‐eye‐tracking studies have found that larger warnings improve recall [8], but there is limited research on how warning size impacts attention. Therefore, eye‐tracking research can provide insight into how warning size impacts attention and interacts with other factors such as standardised packaging [18].

There has also been considerable research using discrete choice experiments (DCEs) to examine the trade‐offs smokers make when considering different characteristics of cigarette packs [21, 22, 23, 24, 25]. DCEs are frequently used in health economics to address policy questions [26], and they can be used to assess tobacco‐control policies [27], particularly in low‐ and middle‐income countries [27, 28]. Moreover, DCEs are a useful technique to understand consumer preferences for not yet marketed products [28]. The DCE methodology relies on Random Utility Theory [28, 29, 30], involving consumer choices for alternative products that vary in key attributes, by assuming that consumers will choose the alternative that offers the greatest value or use. One previous study in Canada used a DCE to investigate the effect of warning size and standardised packaging as attributes influencing intention to try, judgments of taste and harm [23]. The study found that among young females, standardised packaging decreases intentions to try and taste perceptions, and that warning size is important in judging product harm. Another recent DCE study has found similar results among adolescents in Mexico, a country where larger pictorial health warnings and standardised packaging remain to be implemented [25].

This is the first experimental research to examine the impact of standardised packaging and larger health warnings in Colombia. Although the effects are likely to be similar to those observed elsewhere, Colombian policymakers have requested ‘local evidence’ before policy decisions are made [1]. Here, we examine the impact of cigarette pack packaging and health warning size attributes on visual attention, preferences to try and judgements of taste and harm among Colombian smokers and non‐smokers. We used an eye‐tracking experiment and a DCE to fill this evidence gap and provide research that can be used to inform the implementation of these measures in Colombia and worldwide. The full list of study objectives and hypotheses can be found in the pre‐registered study protocol (https://osf.io/jt5n7/).

## METHODS

The study protocol was pre‐registered on the Open Science Framework (https://osf.io/jt5n7/). Ethics approval was obtained from the Ethics Committee at the Universidad Nacional de Colombia (B.VIE‐FCH‐09‐2019).

We conducted an eye‐tracking experiment to examine visual attention to health warnings in comparison to branding information on cigarette packs presented on a computer screen.

We also conducted a DCE to examine the impact of the attributes warning size, packaging and brand type on preferences to try the product, taste perceptions and harm perceptions. The inclusion of brand type attribute responds only to understand the effects of warning size and packaging in more than one brand and for a more realistic DCE.

### Participants

All participants were aged between 18 to 40 years old, lived in Colombia and were fluent in Spanish. Participants were either non‐smokers (smoked fewer than 100 cigarettes in their lifetime and currently not smoking), weekly smokers (smoke at least one cigarette a week, but not every day) or daily smokers (smoke at least five cigarettes a day and smoke within 1 hour of waking). Smokers were not asked to change their smoking habits. We assessed eligibility using an online screening survey before inviting individuals to participate.

Our sample size calculation suggested we required 156 participants for the DCE, 52 in each smoking group to achieve a power of 80% at an α level of 0.05 for two‐sided tests to detect the following effects in the DCE: brand = 0.25, warning size = 0.25, packaging = 0.30, packaging_x_warning size = 0.40 and packaging_x_brand = 0.68. These effect sizes were identified from a previous study [23]. Because the DCE required a larger sample size than the eye‐tracking experiment (75 participants), we used the sample calculation of the DCE for both experiments. For further information regarding the eye‐tracking sample size calculation, please refer to the study protocol (https://osf.io/jt5n7/).

Table 1 shows participant characteristics in the study sample.

**TABLE 1:** Participant demographics and secondary measures

*Variable/Group*	*Total (n = 175)*	*Daily smoker (n = 62)*	*Weekly smoker (n = 58)*	*Non‐smoker (n = 55)*
Female (%)	51	48	50	55
Age	23.11 (4.51)	24.16 (5.18)	21.86 (3.34)	23.34 (4.61)
Socioeconomic status [1, 2, 3, 4, 5, 6, 7, 8]^a^	3	3	3	3
Primarily buy full packs (rather than single cigarettes) (%)	46	65	24	N/A
Breath carbon monoxide (CO) level	5.41 (8.43)	9.74 (0.69)	4.07 (12.24)	1.94 (1.17)
Nicotine dependence (FTND)	1.72 (1.55)	2.43 (1.61)	0.96 (1.05)	N/A
Smoking urges (QSU)	21.32 (7.06)	21.77 (7.37)	20.84 (6.74)	N/A
Quitting Smoking Contemplation Ladder^a^	5	5	5	N/A

Values represent means (standard deviation) unless otherwise stated. FTND = Fagerström Test for Nicotine Dependence; QSU = Questionnaire fo Smoking Urges.

Abbreviations: FTND, Fagerström Test for Nicotine Dependence; QSU, Questionnaire of Smoking Urges.

^a^ Mode.

### Experimental designs, measures and materials

#### Eye‐tracking experiment

We used a mixed model design with warning size (percentage of the cigarette pack covered by the health warning: 30%‐version 1, 30%‐version 2, 50%, 70%) and packaging (standardised packaging, branded packaging) as within‐subject factors; and smoking status (non‐smoker, weekly smoker and daily smoker) as a between‐subject factor. Health warning 30%‐version 1 showed the pictorial on top of the text; meanwhile, 30%‐version 2 showed pictorial and text side‐by‐side. The rest of the warning sizes showed pictorials on top of the text.

Participants viewed cigarette packs individually on screen for 10 seconds each and were asked to remember them. We used an Eyelink II eye‐tracker (SR Research, ON, Canada) to measure eye‐movements and between trials, a gaze‐contingent fixation dot (which allowed for drift correction) was presented. There were six blocks each with eight cigarette pack stimuli. See the study protocol (https://osf.io/jt5n7/) for full details of trial and block randomisation and counterbalancing.

#### Discrete choice experiment

We designed a series of choice sets to examine the impact of three attributes: warning size (30%‐version 1, 70%), packaging (standardised packaging, branded packaging) and brand type (Brand A, Brand B) on preferences to try the product, taste perceptions and harm perceptions. Attribute levels were selected from previous literature [21, 22, 23, 24, 25].

In each trial, participants were shown pairs of cigarette packs and were asked to choose one of them. Participants' preferences were, therefore, conditional on accepting one of the two cigarette packs, a trial‐type hereafter referred to as ‘conditional trials’. We also included ‘unconditional trials’ where participants had an option to select ‘neither of these’ to represent a real‐world decision‐making process, where consumers have the choice not to buy.

For each cigarette pack, we manipulated three features, each of which had two options (feature‐levels): (i) packaging (branded, standardised); (ii) warning size (30%, 70%); (iii) brand name (Brand A, Brand B). This resulted in 2^3^ = 8 different cigarette packs. We used the full factorial design, optimal for estimating the main effects of the features and their two‐factor interactions: packaging_x_warning size and packaging_x_brand name [23]. The optimal design included each of the cigarette pack pairs (i.e., choice sets) where two of the three features differed between the packs [31, 32], resulting in 12 choice sets. The list of all choice sets used is reported in the Supporting Information (Table S1).

Participants completed three blocks, each corresponding to one of the outcomes: preferences to try; taste perceptions; harm perceptions. Blocks were presented in random order and each included all of our 12 choice sets with the conditional and unconditional trials intermixed within each block, such that there were a total of 72 choice sets presented. The sequence within the blocks and within the choice sets was presented randomly to each participant.

#### Cigarette pack stimuli

For the eye‐tracking experiment, cigarette pack stimuli used six of the most popular Colombian tobacco brands [33]. Standardised packs were designed specifically for this study following the WHO and United Kingdom (UK) Government guidelines on standardised packaging [34, 35]. Health warnings comprised the six Colombian health warnings introduced in 2018 depicting the risks of smoking for death, pancreatic cancer, heart damage, anxiety, the unborn child and risks of second‐hand smoke. Health warnings showed the pictorial on the top and the text underneath and were cropped so together they filled 30% (30%‐version 1), 50% or 70% of the pack. We also included an additional 30% condition (30%‐version 2) where the text and pictorial were side‐by‐side to reflect current practice in Colombia. By combining each of these elements we designed a total of 288 stimuli.

**FIGURE 1:**
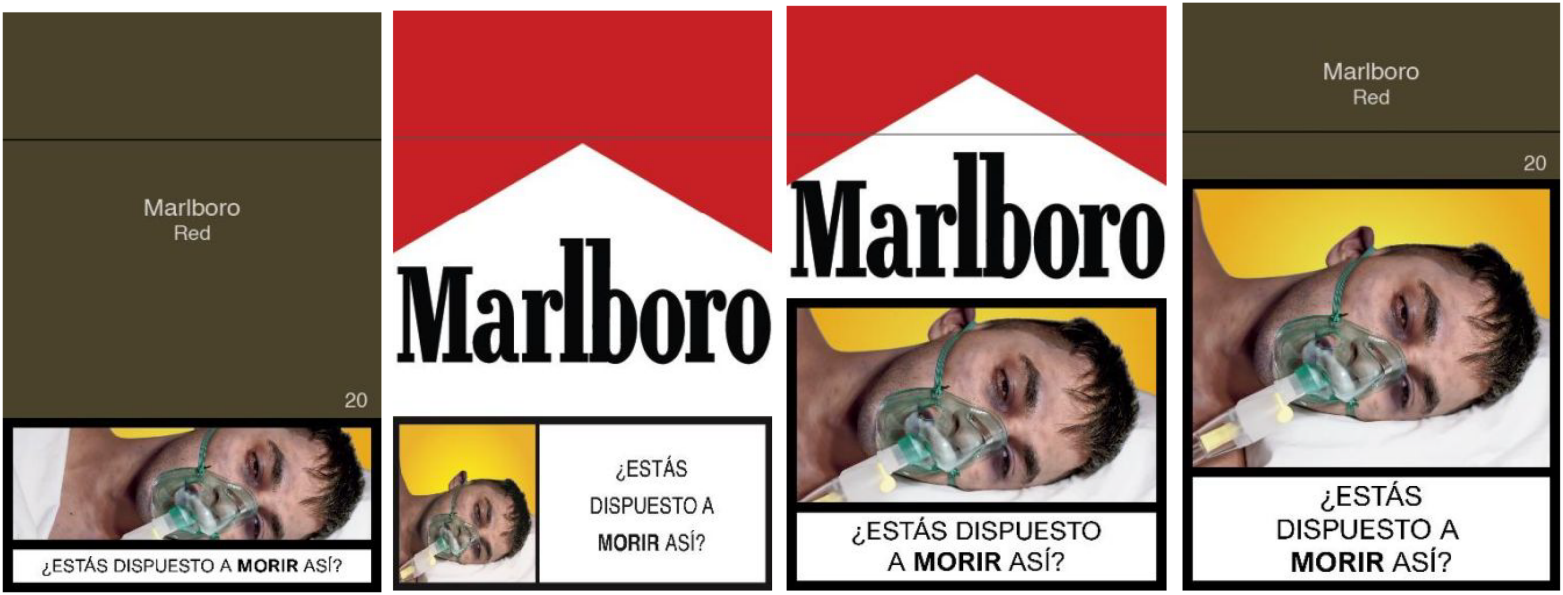
Examples of the cigarette packing stimuli used in this study: from left to right 1) standardised 30% version‐1, 2) branded 30% version‐2 (current practice), 3) branded 50% and 4) standardised 70%

For the DCE, we used eight stimuli created for the eye‐tracking experiment. The choice sets showed the 30%‐version 1 and 70% health warning designs in both standardised and branded packaging. We used a single health warning (smoking and death; see Fig. 1). We only used the two most popular brands in Colombia [33], Marlboro and Lucky Strike, although we refer to these as ‘Brand A’ and ‘Brand B’ here (not necessarily in that order), as we consider that our results may be commercially sensitive.

The study protocol (https://osf.io/jt5n7/) includes several examples of the stimuli created for this study.

#### Primary outcome measures

For the eye‐tracking experiment, our primary outcome measure was the difference in the number of fixations on the warning as compared with the branding for all trials combined. We also measured the duration of fixations on the two regions and the location of the first fixation (again reported as difference scores).

For the DCE, the outcome measures, which have been used previously [23], were: preferences to try (‘Which one of these brands would you rather try?’), taste perceptions (‘Which one of these brands do you think would taste better?’) and harm perceptions (‘Which one to these brands do you think would be less harmful?’).

### Procedure

Eligible participants attended a single testing session lasting ~45 minutes. Participants provided informed consent and completed a breath carbon monoxide (CO) measure, demographic questions related to their gender, age and socioeconomic status (from 1 ‘most deprived’ to 8 ‘least deprived’); and, if smokers, the Colombian Spanish versions of the Fagerström Test for Nicotine Dependence (FTND) [36], the Brief Questionnaire of Smoking Urges (QSU‐Brief) [37] and the Quitting Smoking Contemplation Ladder [38]. Smokers also reported whether they primarily buy whole packaged cigarettes or single cigarettes (a practice which is prohibited but poorly enforced and therefore common in Colombia) [39].

Participants then completed the eye‐tracking experiment, followed by the DCE. Participants were then fully debriefed and reimbursed $25, 000 COP (equivalent to £6) for their time.

### Statistical analysis

For the eye‐tracking experiment, the principal analysis was a 4 (health warning size: 30%‐version 1, 30%‐version 2, 50%, 70%) × 2 (packaging: branded, standardised) × 3 (smoking status: non‐smoker, weekly smoker, daily smoker) and the interaction effects warning size_×_packaging, warning size_×_smoking status and packaging_×_smoking status mixed model multivariate analysis of variance (MANOVA) of the three outcome variables.

We used conditional logit models to analyse the DCE. The goodness‐of‐fit of the models was evaluated using the Akaike Information Criterion (AIC). The best‐fitted model for each outcome measure was defined as the one that minimised the AIC. This led to removing the interaction packaging_x_waning size term to optimize parsimony. The models M1 and M2 present the feature and interaction terms retained in the best‐fitted conditional and unconditional preference models, respectively. Results of other fitted models (non‐optimal) including the interaction between packaging and warning size are presented in the Supporting Information (Figure S1 and Tables S8 and S9). In both models, each choice set includes two alternative cigarette packs, in addition to the ‘neither‐of these’ choice in Model M2 only.

Model M1 (conditional preference trials):

$$U_{j}=\beta_{1}\cdot {\text{PACKAGING}}_{j}+\beta_{2}\cdot {\text{BRAND}}_{j}+\beta_{3}\cdot {\text{SIZE}}_{j}+\beta_{12}\cdot {\text{PACKAGING}}_{j}\cdot {\text{BRAND}}_{j}+\varepsilon_{j}.$$

Model M2 (unconditional preference trials):

$$U_{j}=\alpha +\beta_{1}\cdot {\text{PACKAGING}}_{j}+\beta_{2}\cdot {\text{BRAND}}_{j}+\beta_{3}\cdot {\text{SIZE}}_{j}+\beta_{12}\cdot {\text{PACKAGING}}_{j}\cdot {\text{BRAND}}_{j}+\varepsilon_{j}.$$

*U*_*j*_ denotes the use for alternative *j*, *β*_*1*_ represents the effect of the standardised packaging category, compared with the branded packaging (reference level), *β* *_2_* represents the effect of the Brand B, compared with the Brand A (reference level), *β*_*3*_ is the effect of a 10% increase in the warning size, *β*_*12*_ denotes the interaction effect between the packaging and brand features, and *α* is the alternative specific constant (ASC) representing the intercept of use in Model M2. The parameter *α* is set to be zero for the ‘neither‐of‐these’ choice, and non‐zero for the cigarette pack alternatives, as it reflects the baseline use of the cigarette pack alternatives (i.e., the use corresponding to the features at their reference levels). ε_j_ represents the random error associated with the alternative *j*. We examined the estimated effects sizes between these two models to understand the influence of giving participants the option ‘neither‐of‐these’.

We have adjusted the models M1 and M2 for smoking status (non‐smoker, weekly smoker and daily smoker), age (centred at its mean value, 23 years) and gender to examine the effects of these variables on the attributes for each of our outcome measures. Specifically, we explored the following interactions: packaging_×_smoking status, packaging_×_gender, packaging_×_age, warning size_x_smoking status, warning size_×_age, brand_×_smoking status, brand_×_age and brand_×_gender.

Results were expressed using ORs, 95% CI, and the exact *P* values of the considered effects. Because we investigate three outcomes, we have used the Bonferroni adjustment [40] to account for multiple testing. In a sensitivity analysis, we used the adjusted significance level of 0.017 to assess the effect of considered attributes and their interactions on each of the three outcomes. All analyses were conducted using R, version 3.6.1.

## RESULTS

### Eye‐tracking experiment

**FIGURE 2:**
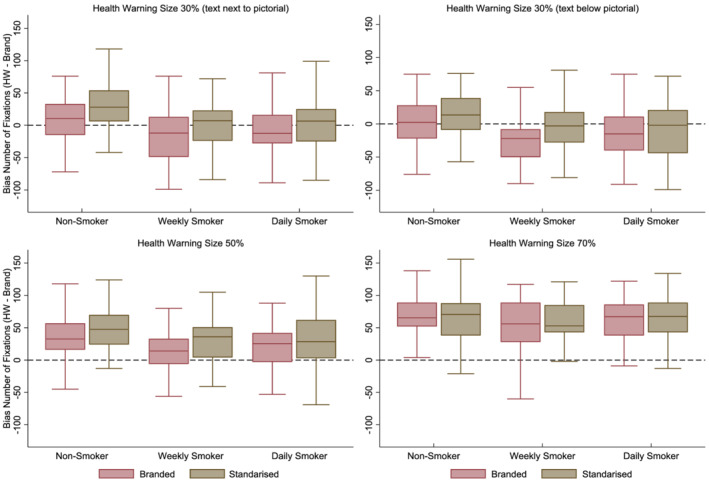
Bias in number of fixations (number of fixations toward the health warning [HW] minus the brand) for branded packaging (in red) and standardised packaging (in olive colour) among non‐smokers, weekly smokers, and daily smokers. On the top left for health warning size 30%‐version 1 (text next to pictorial), on the top right for health warning size 30%‐version 2 (text below pictorial), on the bottom left for health warning size 50% and on the bottom right for health warning size 70%. Positive values (above the dashed line) mean more visual attention (i.e., more fixations) toward the health warning in comparison to the branding

Two participants failed the eye‐tracking calibration; therefore, a total of 173 participants completed this part of the study. Below we report the multivariate and univariate results for the bias in the number of fixations and these data are presented in Fig. 2. There were no violations of main MANOVA assumptions. See Supporting Information Table S2 for complete univariate results for the bias in duration of fixations and the first fixations.

#### Visual attention to warnings is greater on standardised than branded packs

There was clear evidence of the effect of packaging (multivariate: Wilks' Lambda = 0.71, *F*[3,167] = 22.87, *P* < 0.001, *η*_*p*_^*2*^ = 0.29; univariate: *F*[1,169] = 56.73, *P* < 0.001, *η*_*p*_^*2*^ = 0.25), such that there was greater visual attention to health warnings (compared with branding) when they were placed on standardised (M = 27.26, SD = 38.66) than branded packs (M = 16.73, SD = 37.53).

#### Visual attention is greater to larger health warnings

There was greater attention to larger warnings (multivariate: Wilks’ Lambda = 0.11, *F*[9,161] = 147.17, *P* < 0.001, *η*_*p*_^*2*^ = 0.89; univariate: *F*[1,169] = 516.61, *P* < 0.001, *η*_*p*_^*2*^ = 0.75) such that the mean bias between branding and health warnings was M = 2.72 (SD = 40.89) for the 30%‐version 1 warning, M = −6.72 (SD = 37.70) for the 30%‐version 2 warning, M = 28.78 (SD = 37.54) for the 50% warning and M = 63.18 (SD = 36.24) for the 70% warning (note that higher scores represent greater attention to warnings over branding).

#### As health warning size increased, the difference in visual attention to warnings on standardised and branded packaging decreased

There was also an interaction between packaging and warning size (multivariate: Wilks' Lambda = 0.8, *F*[9,161] = 4.44, *P* < 0.001, *η*_*p*_^*2*^ = 0.2; univariate: *F*[3,169] = 5.48, *P =* 0.001, *η*_*p*_^*2*^ = 0.03). We observed greater differences in the bias in number of fixations to warnings between standardised and branded packs when the warnings were smaller (standardised vs branded packaging: 30%‐version 1 MD = 13.84, SE = 2.43, 95% CI = [9.04,18.63]; 30%‐version 2 MD = 12.31, SE = 2.19, 95% CI = [7.98,16.63]; 50% MD = 11.47, SE = 2.28, 95% CI = [6.97,15.97]; and 70% MD = 3.56, SE = 2.07, 95% CI = [−0.52,7.64]).

#### Non‐smokers visually attend toward the warnings more than smokers, but as warning size increased these differences decreased

Visual attention to the warnings versus branding differed by smoking status (multivariate: Wilks' Lambda = 0.9, *F*[6,334] = 2.92, *P* = 0.009, *η*_*p*_^*2*^ = 0.05; univariate: *F*[2,169] = 8.31, *P* < 0.001, *η*_*p*_^*2*^ = 0.09). Both, daily smokers (MD = −16.47, SE = 5.63; 95% CI = [−27.58, −5.36]) and weekly smokers (MD = −22.76, SE = 5.77, 95% CI = [−34.14, −11.37]) made fewer fixations to health warnings than non‐smokers. There was no clear evidence for a difference between daily smokers and weekly smokers (MD = −6.28, SE = 5.57, 95% CI = [−17.28,4.72]).

There was evidence for the interaction between smoking status and health warning size (multivariate: Wilks' Lambda = 0.83, *F*[18,322] = 1.74, *P* = 0.03, *η*_*p*_^*2*^ = 0.09; univariate: *F*[2,169] = 2.87, *P* = 0.01, *η*_*p*_^*2*^ = 0.03). Figure 2 demonstrates that there were smaller differences in the bias in number of fixations between smokers and non‐smokers for larger warning sizes.

There was no clear evidence for the interaction effect between smoking status and packaging (multivariate: Wilks' Lambda = 0.95, *F*[6,334] = 1.48, *P* = 0.18, *η*_*p*_^*2*^ = 0.03; univariate: *F*[2,169] = 3.28, *P* = 0.05, *η*_*p*_^*2*^ = 0.04).

### Discrete choice experiment

The final models presented below excluded the packaging_x_warning size interaction, because there was no clear evidence of its effect. The results for the suboptimal models (including this interaction) can be found in Supporting Information Fig. S1 and Tables S8 and S9. Results of the final models with Bonferroni adjustment (Supporting Information Fig. S2) were similar to the models without adjustment, therefore, we decided to communicate the latter. Supporting Information Table S3 shows the percentage of participants who chose ‘neither‐of‐these’ option in the unconditional trials.

**FIGURE 3:**
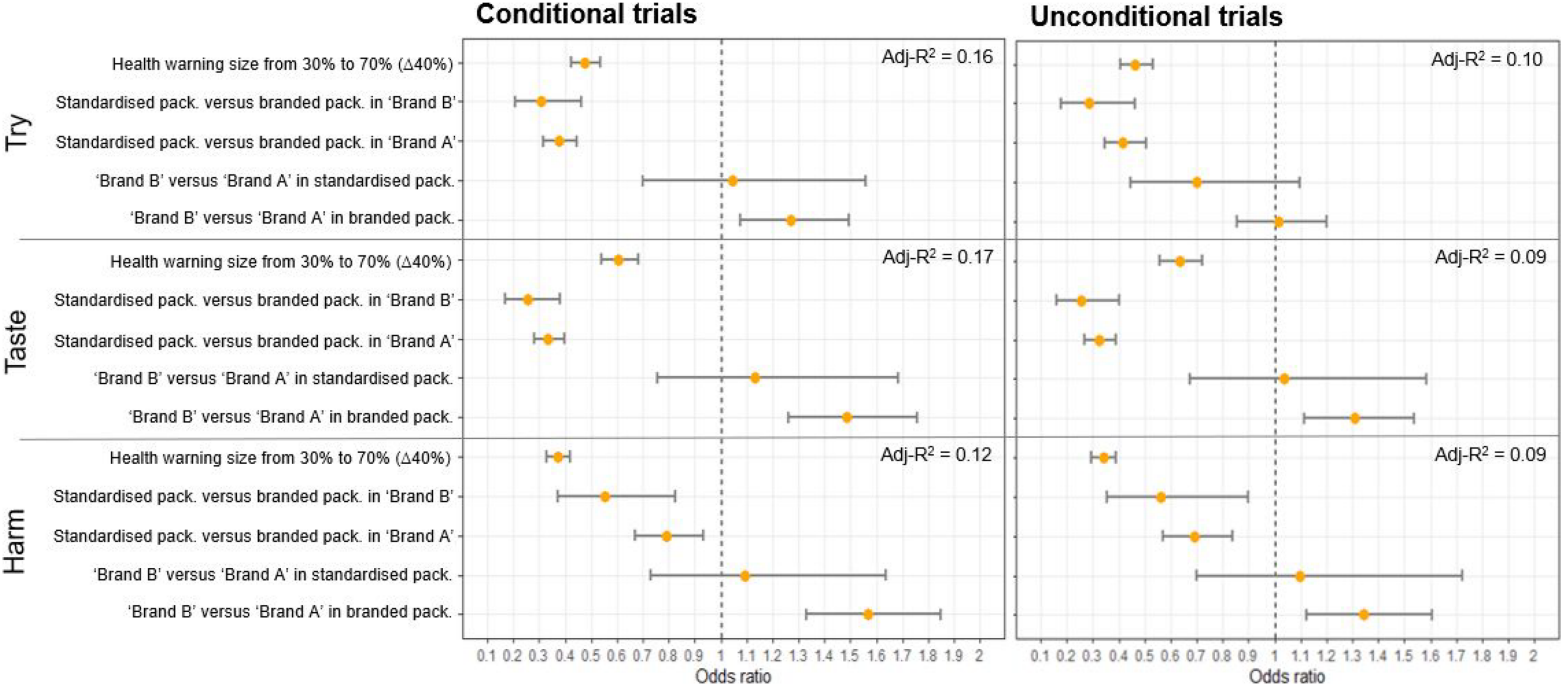
Odds ratios and 95% confidence intervals for the conditional and unconditional trials for preferences to try, taste perceptions and harm perceptions. Odds ratios less than 1 (i.e., lying to the left of the dashed line) indicate that the corresponding attribute is associated with lower preferences to try, lower taste perceptions and higher harm perceptions. Odds ratios larger than 1 (i.e., lying to the right of the dashed line) indicate that the corresponding attribute is associated with higher preferences to try, higher taste perceptions and lower harm perceptions. The confidence intervals that intersect with the dashed line indicate there is no evidence the corresponding attribute is associated with the preferences

Figure 3 shows the results for unadjusted conditional and unconditional models, see Supporting Information Tables S4 and S5 for more detailed results.

#### Preference to try

In the unconditional trials, participants chose one of the two alternative packs in 65% of trials, compared with choosing ‘neither of these’ in the remaining 35% of trials.

Increasing the warning size by 10% was associated with reduced preferences to try for the conditional trials (OR = 0.83, 95% CI = [0.81,0.86], *P* < 0.001) and the unconditional trials (OR = 0.83, 95% CI = [0.80,0.85], *P* < 0.001). This means that increasing the warning size from 30% to 70% (∆40%) reduced preferences to try by 52% for conditional trials (OR = 0.48, 95% CI = [0.42,0.54]) and 54% for unconditional trials (OR = 0.46, 95% CI = [0.40,0.53]).

For Brand A, standardised packaging (compared with branded packaging) was associated with 63% reduced preferences to try in conditional trials (OR = 0.37, 95% CI = [0.32,0.44], *P* < 0.001) and 58% in unconditional trials (OR = 0.42, 95% CI = [0.34,0.51], *P* < 0.001). For Brand B, standardised packaging reduced preferences to try by 69% in conditional trials (OR = 0.31, 95% CI = [0.21,0.46], *P* < 0.001) and 71% in unconditional trials (OR = 0.29, 95% CI = [0.18,0.46], *P* < 0.001).

When in branded packaging, Brand B (compared with Brand A) was associated with increased preferences to try in conditional trials (OR = 1.27, 95% CI = [1.07,1.50], *P* *= 0.01*). There was no evidence of such an association in unconditional trials (OR = 0.7, 95% CI = [0.44,1.10], *P* = 0.88). There were no differences between brands when packs were standardised.

#### Perceptions of product taste

In the unconditional trials, participants chose one of the presented two alternative packs in 66% of the trials, whereas ‘neither of these’ was selected in 34% of the trials.

Increasing the size of the health warning by 10% was associated with reduced taste perceptions (conditional trials, OR = 0.88, 95% CI = [0.86,0.91], *P* < 0.001; unconditional trials, OR = 0.89, 95% CI = [0.86,0.92], *P* < 0.001). This means that increasing the warning size from 30% to 70% (∆40%) reduced taste perceptions by 39% for conditional trials (OR = 0.61, 95% CI = [0.54,0.68]) and 37% for unconditional trials (OR = 0.63, 95% CI = [0.86,0.92]).

For Brand A, standardised packaging (compared with branded packaging) was associated with 67% reduced taste perceptions in conditional trials (OR = 0.33, 95% CI = [0.28,0.40], *P* < 0.001) and 69% in the unconditional trials (OR = 0.32, 95% CI = [0.27,0.39], *P* < 0.001). For Brand B standardised packaging reduced taste perceptions by 75% in both conditional (OR = 0.25, 95% CI = [0.17,0.38], *P* < 0.001) and unconditional trials (OR = 0.25, 95% CI = [0.16,0.40], *P* < 0.001).

Brand B (compared with Brand A) was associated with increased taste perceptions in branded packages for both conditional trials (OR = 1.49, 95% CI = [1.26,1.76], *P* < 0.001) and unconditional trials (OR = 1.31, 95% CI = [1.12,1.54], *P* < 0.001). There were no differences between brands for standardised packs.

#### Perceptions of product harm

In the unconditional trials, participants chose one of the presented two alternative packs in 56% of the trials, whereas ‘neither of these’ was selected in 44% of trials.

Increasing the warning size by 10% was associated with increased harmful perceptions (conditional trials, OR = 0.78, 95% CI = [0.76,0.80], *P* < 0.001; unconditional trials, OR = 0.76, 95% CI = [0.74,0.79], *P* < 0.001). This means that increasing the warning size from 30% to 70% (∆40%) increased harm perceptions by 63% for conditional trials (OR = 0.37, 95% CI = [0.33,0.42]) and 66% for unconditional trials (OR = 0.34, 95% CI = [0.30,0.39]).

For Brand A, standardised packaging (compared with branded packaging) was associated with 21% increased harm perceptions in the conditional trials (OR = 0.79, 95% CI = [0.67,0.93], *P* < 0.001) and 31% in the unconditional trials (OR = 0.69, 95% CI = [0.57,0.84], *P* < 0.001). For Brand B standardised packaging increased harm perceptions by 45% in the conditional trials (OR = 0.55, 95% CI = [0.37,0.83], *P* < 0.001) and 44% in the unconditional trials (OR = 0.56, 95% CI = [0.35,0.90], *P* < 0.001).

Brand B (compared with Brand A) was associated with reduced harm perceptions in branded packages for both conditional trials (OR = 1.57, 95% CI = [1.33,1.85]) and unconditional trials (OR = 1.34, 95% CI = [1.12,1.61]). There were no differences between brands for standardised packs.

#### Models adjusted for smoking status, age and gender

See Supporting Information Tables S6 and S7 for the full results of the adjusted models for conditional and unconditional trials.

Compared with daily smokers, non‐smokers had reduced preferences to try standardised packaging (conditional trials, OR = 0.48, 95% CI = [0.34,0.67], *P* < 0.001; unconditional trials, OR = 0.62, 95% CI = [0.38,1.01], *P* = 0.061). Similar findings were obtained for taste perceptions (conditional trials, OR = 0.53, 95% CI = [0.38,0.76], *P* < 0.001); unconditional trials, OR = 0.40, 95% CI = [0.26,0.60], *P* < 0.001). However, there was no evidence for a difference in harm perceptions for standardised packaging between non‐smokers and daily smokers (conditional trials, OR = 1.11, 95% CI = [0.95,1.27], *P* = 0.49; unconditional trials, OR = 1.29, 95% CI = [0.92,1.80], *P* = 0.142). There were no meaningful differences between weekly and daily smokers for any of these outcomes.

Larger warnings reduced preferences to try to a greater extent among non‐smokers compared with daily smokers in conditional trials (OR = 0.87, 95% CI = [0.81,0.95], *P* = 0.001, per 10% increase in warning size); but there was no clear evidence for this effect in the unconditional trials (OR = 1.08, 95% CI = [0.98,1.19], *P* = 0.15). Larger warnings also increased harm perceptions to a greater extent among non‐smokers compared with daily smokers (conditional trials, OR = 0.90, 95% CI = [0.84,0.98], *P* = 0.01; unconditional trials, OR = 0.91, 95% CI = [0.84,0.98], *P* = 0.03). There was no clear evidence of a difference in taste perceptions between non‐smokers and daily smokers at different warning sizes. There were no meaningful differences between weekly and daily smokers for any of these outcomes.

In the conditional trials, there was some evidence that males had higher harm perceptions for standardised packs than females (OR = 0.78, 95% CI = [0.61,0.98], *P* = 0.03). There were no other meaningful differences regarding gender and packaging or warning size.

For standardised packaging, increased age was associated with: increased preferences to try (conditional trials, OR = 1.04, 95% CI = [1.01,1.06]; unconditional trials, OR = 1.04, 95% CI = [1.01,1.08], *P* = 0.02 per one‐year of age difference); higher taste perceptions only in the conditional trials (conditional trials, OR = 1.05, 95% CI = [1.02,1.08], *P* = 0.003; unconditional trials, OR = 1.02, 95% CI = [0.98,1.06], *P* = 0.21); and lower harm perceptions (conditional trials, OR = 1.05, 95% CI = [1.03,1.08], *P* < 0.001; unconditional trials, OR = 1.54, 95% CI = [1.02,1.09], *P* = 0.004).

## DISCUSSION

Covering just 30% of the cigarette pack, tobacco health warnings in Colombia fail to meet WHO FCTC recommendations for warning size. Previous research has consistently shown that larger health warnings and standardised packaging encourages attention to warnings, increases knowledge of risks and changes smoking behaviour [7, 8, 10, 12, 13, 20, 27]. Our research demonstrates that these tobacco control measures are also likely to be effective in Colombia, an important finding for local policymakers.

In our eye‐tracking experiment, we observed greater visual attention to larger warnings, an effect we saw for non‐smokers and smokers alike. This is important, because attention is the necessary first step in warning processing and eventual behaviour change [12]. Supporting previous DCEs [22, 23, 25], increasing health warning size also influenced choice; participants were less likely to select packs with warnings covering 70% (vs 30%) as those they would like to try, and more likely to select them as those that would taste worse and would be more harmful. These results contribute to the current evidence of how larger warnings might improve attention and interact with standardised packing [18].

Supporting our previous work [9, 19, 20], we find that standardised packaging increases visual attention to small (30%) warnings, particularly among non‐smokers. However, because health warning size increased, the difference in visual attention to warnings between standardised and branded packs decreased, as did differences between non‐smokers and smokers. This raises the following question: is standardised packaging required if larger health warnings are introduced? Although our eye‐tracking findings suggest that 70% health warnings are sufficient in increasing attention to warnings, the results from the DCE showed that standardised packaging is also an important policy in reducing preferences to try, taste preferences and increasing harm perceptions, regardless of warning size as we saw no interaction between packaging type and warning size. These results were consistent in both conditional and unconditional DCE trials and align with previous research conducted elsewhere [7, 11, 23, 25]. However, future studies should investigate the interaction between warning size and standardisation in more detail as other studies have found mixed results [23, 25]. Furthermore, we found that standardised packaging reduced brand differentiation, such that participants were less likely to select Brand A over Brand B as that which would taste better or be less harmful when both were in standardised packs.

To our knowledge, this is the first experiment in Colombia testing the effects of standardised packaging and larger health warnings with these innovative methodologies (i.e., eye‐tracking and DCE) and our research supports and replicates previous work conducted elsewhere [7, 8, 10, 12, 20, 27]. The implementation of these policies might contribute to reducing the burden that tobacco places on the Colombian economy and health care system [41]. Although governments may require, or at least welcome, ‘local’ evidence on the effects of tobacco control policies, such as larger health warnings and standardised packaging (as per the Colombian Ministry of Health) [1], our research demonstrates that these policies are likely to have similar effects cross‐culturally.

Our research has some limitations. First, we used two‐dimensional stimuli and responses may vary from these stimuli to actual cigarette packs. Second, our participants are relatively young and other research should confirm whether similar effects are observed in older adults and adolescents, and other smoking groups (e.g., heavier smokers or users of other tobacco products). Finally, although we measured several different outcomes using two experimental designs, we were not able to measure the impact of these policies on actual behaviour, or longer‐term effects. Indeed, the Colombian tobacco retail environment may limit the effectiveness of these policies. Although prohibited by law, many smokers do not buy whole packs of cigarettes (24% of daily smokers and 65% of weekly smokers in our study bought single cigarettes) and are less likely to regularly view the warnings on packs. Therefore, in addition to implementing these policies, we suggest that there should be greater enforcement of tobacco retail regulations, alongside mass media campaigns about the risks of smoking and information about how to quit.

Our research supports the inclusion of both larger health warnings and standardised packaging. We suggest that combined, these measures increase visual attention to the warnings and may reduce the demand for cigarettes and misleading perceptions about cigarette products. As our research in Colombia supports similar research conducted elsewhere, we suggest that these effects are likely to be the same cross‐culturally.

## DECLARATION OF INTERESTS

None.

## AUTHOR CONTRIBUTIONS

**Carlos Sillero‐Rejon**: Conceptualization; design the experiments; data curation; formal analysis; methodology; project administration; resources; software; supervision; visualization; writing – original draft; writing – review & editing. **Osama Mahmoud**: Conceptualization; design the experiments, data curation; formal analysis; methodology; software; visualization; writing – review & editing. **Ricardo M Tamayo**: Conceptualization; data curation; formal analysis; methodology; project administration; software; supervision; visualization. **Alvaro Arturo Clavijo‐Alvarez**: Conceptualization; data curation; formal analysis; methodology; project administration; supervision. **Sally Adams**: Conceptualization; data curation; formal analysis; methodology; software; visualization. **Olivia Maynard**: Conceptualization; data curation; formal analysis; funding acquisition; methodology; project administration; resources; software; supervision; visualization; writing – review & editing. The first two authors are co‐first authors (listed in alphabetic order).

## Supporting information


**Table S1** Choice sets of the optimal design for estimating main effects and two‐factor interactions.
**Table S2** Univariate results for bias number of fixation, bias duration of fixation and bias first fixation.
**Table S3** Percentage of participants who chose ‘neither of these’ option in the unconditional trials in the discrete choice experiment.
**Table S4** Conditional choice sets discrete choice experiment results, unadjusted.
**Table S5** Unconditional choice sets discrete choice experiment results, unadjusted.
**Table S6** Conditional choice sets discrete choice experiment results adjusted for smoking status, gender and age.
**Table S7** Unconditional choice sets discrete choice experiment results adjusted to smoking status, gender and age.
**Figure S1** Conditional and unconditional results of suboptimal models.
**Figure S2** Conditional and unconditional results models with Bonferroni adjustment.
**Table S8** Suboptimal model—conditional choice sets discrete choice experiment results, unadjusted.
**Table S9** Suboptimal model—unconditional choice sets discrete choice experiment results, unadjusted.

## Data Availability

The data that form the basis of the results presented in this manuscript are available from the University of Bristol Research Data Repository (http://data.bris.ac.uk/data/), DOI: 10.5523/bris.y1t0fiupaioe29uqgnermco0z.
