# Supplementary material for: Standardised packs and larger health warnings: visual attention and perceptions among Colombian smokers and non‐smokers
Source: Addiction. 2022 Jan 8;117(6):1737–47. doi: 10.1111/add.15779 (PMC9306697; doi:10.1111/add.15779)
Supplement: Supplementary file 1 — Table S1 Choice sets of the optimal design for estimating main effects and two‐factor interactions. Table S2 Univariate results for bias number of fixation, bias duration of fixation and bias first fixation. Table S3 Percentage of participants who chose ‘neither of these’ option in the unconditional trials in the discrete choice experiment. Table S4 Conditional choice sets discrete choice experiment results, unadjusted. Table S5 Unconditional choice sets discrete choice experiment results, unadjusted. Table S6 Conditional choice sets discrete choice experiment results adjusted for smoking status, gender and age. Table S7 Unconditional choice sets discrete choice experiment results adjusted to smoking status, gender and age. Figure S1 Conditional and unconditional results of suboptimal models. Figure S2 Conditional and unconditional results models with Bonferroni adjustment. Table S8 Suboptimal model—conditional choice sets discrete choice experiment results, unadjusted. Table S9 Suboptimal model—unconditional choice sets discrete choice experiment results, unadjusted. [file ADD-117-1737-s001.docx]

**Supplementary Table 1**: Choice sets of the optimal design for estimating main effects and two-factor interactions.

| **Set** | **Option 1** | **Option 2** | **Un-coded option 1** | **Un-coded option 2** |
| --- | --- | --- | --- | --- |
| 1 | $000$ | $011$ | Brand A, standardised, 30% | Brand A, branded, 70% |
| 2 | $001$ | $010$ | Brand A, standardised, 70% | Brand A, branded, 30% |
| 3 | $100$ | $111$ | Brand B, standardised, 30% | Brand B, branded, 70% |
| 4 | $101$ | $110$ | Brand B, standardised, 70% | Brand B, branded, 30% |
| 5 | $000$ | $101$ | Brand A, standardised, 30% | Brand B, standardised, 70% |
| 6 | $001$ | $100$ | Brand A, standardised, 70% | Brand B, standardised, 30% |
| 7 | $111$ | $010$ | Brand B, branded, 70% | Brand A, branded, 30% |
| 8 | $110$ | $011$ | Brand B, branded, 30% | Brand A, branded, 70% |
| 9 | $000$ | $110$ | Brand A, standardised, 30% | Brand B, branded, 30% |
| 10 | $001$ | $111$ | Brand A, standardised, 70% | Brand B, branded, 70% |
| 11 | $100$ | $010$ | Brand B, standardised, 30% | Brand A, branded, 30% |
| 12 | $101$ | $011$ | Brand B, standardised, 70% | Brand A, branded, 70% |

**Supplementary Table 2.** Univariate Results for Bias Number of Fixation, Bias Duration of Fixation and Bias First Fixation.

| **Factor** | **Measure** | **F** | **Sig.** | **Partial Eta Squared** | **Observed Power** |
| --- | --- | --- | --- | --- | --- |
| **Packaging** | Bias Number of Fixation | 56.73 | <0.001 | 0.25 | 1.00 |
|  | Bias Duration of Fixation | 25.67 | <0.001 | 0.13 | 1.00 |
|  | Bias First Fixation | 0.29 | 0.59 | <0.001 | 0.08 |
| **Size** | Bias Number of Fixation | 516.61 | <0.001 | 0.75 | 1.00 |
|  | Bias Duration of Fixation | 419.02 | <0.001 | 0.71 | 1.00 |
|  | Bias First Fixation | 533.60 | <0.001 | 0.76 | 1.00 |
| **Smoking Status** | Bias Number of Fixation | 8.31 | <0.001 | 0.09 | 0.96 |
|  | Bias Duration of Fixation | 6.98 | <0.001 | 0.08 | 0.92 |
|  | Bias First Fixation | 2.92 | 0.06 | 0.03 | 0.56 |
| **Packaging * Size** | Bias Number of Fixation | 5.48 | <0.001 | 0.03 | 0.94 |
|  | Bias Duration of Fixation | 4.20 | 0.01 | 0.02 | 0.86 |
|  | Bias First Fixation | 3.04 | 0.03 | 0.02 | 0.71 |
| **Packaging * Smoking Status** | Bias Number of Fixation | 3.28 | 0.04 | 0.04 | 0.62 |
|  | Bias Duration of Fixation | 2.99 | 0.05 | 0.03 | 0.57 |
|  | Bias First Fixation | 0.97 | 0.38 | 0.01 | 0.22 |
| **Size * Smoking Status** | Bias Number of Fixation | 2.87 | 0.01 | 0.03 | 0.89 |
|  | Bias Duration of Fixation | 4.15 | <0.001 | 0.05 | 0.98 |
|  | Bias First Fixation | 0.38 | 0.90 | <0.001 | 0.16 |

PACKAGING (standardised packaging versus branded packaging); Size (percentage of the cigarette pack covered by the health warning: 30%-version 1 versus 30%-version 2 versus 50% versus 70%) as within-subject factors and smoking status (non-smoker versus weekly smoker versus daily smoker) as a between-subject factor. Bias in the number of fixations towards the warning as compared with the PACKAGING (calculated as a difference score); bias in the duration of the fixations and the bias in the number of times that the two regions were the location of the first fixation.

**Supplementary Table 3**: Percentage of participants who chose “neither of these” option in the unconditional trials in the Discrete Choice Experiment

| **Outcome** | **Percentage** |
| --- | --- |
| Preferences to try | **35%** |
| Taste | **34%** |
| Less harmful | **44%** |
| Total | **38%** |

**Supplementary Table 4.** Conditional Choice Sets Discrete Choice Experiment Results – unadjusted.

| **OUTCOME** | **ATTRIBUTE** | **coef** | **OR** | **se** | **z** | **p** |
| --- | --- | --- | --- | --- | --- | --- |
| **TRY** | Packaging (Ref.: Branded pack.) | -0.98 | 0.37 | 0.09 | -11.42 | <0.001 |
|  | Brand (Ref.: Brand A) | 0.24 | 1.27 | 0.08 | 2.81 | 0.01 |
|  | Size (10% increase) | -0.19 | 0.83 | 0.02 | -12.26 | <0.001 |
|  | Packaging:brand | -0.19 | 0.82 | 0.12 | -1.61 | 0.11 |
|  | Adj-R2 | 0.16 | | | | |
|  | AIC | 2433.21 | | | | |
|  | BIC | 2455.81 | | | | |
| **TASTE** | Packaging (Ref.: Branded pack.) | -1.10 | 0.33 | 0.09 | -12.67 | <0.001 |
|  | Brand (Ref.: Brand A) | 0.40 | 1.49 | 0.08 | 4.69 | <0.001 |
|  | Size (10% increase) | -0.12 | 0.88 | 0.01 | -8.34 | <0.001 |
|  | Packaging:brand | -0.28 | 0.76 | 0.12 | -2.31 | 0.02 |
|  | Adj-R2 | 0.17 | | | | |
|  | AIC | 2405.22 | | | | |
|  | BIC | 2427.82 | | | | |
| **LESS HARMFUL** | Packaging (Ref.: Branded pack.) | -0.23 | 0.79 | 0.08 | -2.82 | <0.001 |
|  | Brand (Ref.: Brand A) | 0.45 | 1.57 | 0.08 | 5.33 | <0.001 |
|  | Size (10% increase) | -0.25 | 0.78 | 0.02 | -16.19 | <0.001 |
|  | Packaging:brand | -0.36 | 0.70 | 0.12 | -2.95 | <0.001 |
|  | Adj-R2 | 0.16 | | | | |
|  | AIC | 2433.21 | | | | |
|  | BIC | 2455.81 | | | | |

Try – preferences to try; Taste - better taste perceptions; Less harmful – less harmful perceptions. Packaging - the effect of the standardised packaging category (versus branded packaging) with the ‘Brand A’; Brand - the effect of the ‘Brand B’ (versus ‘Brand A’) standardised packaging; Size - the effect of 10% increase in the warning size; Packaging ×Brand - the effect of standardised packaging (versus branded packaging) with the ‘Brand B. Adj-R2 - R-Squared value adjusted by the number of estimated coefficients; AIC -n Akaike information criterion; BIC – Bayesian information criterion. **Supplementary Table 5.** Unconditional Choice Sets Discrete Choice Experiment Results – unadjusted.

| **OUTCOME** | **ATTRIBUTE** | **coef** | **OR** | **se** | **z** | **p** |
| --- | --- | --- | --- | --- | --- | --- |
| **TRY** | ASC | -0.01 | 0.99 | 0.11 | -0.10 | 0.92 |
|  | Packaging (Ref.: Branded pack.) | -0.87 | 0.42 | 0.10 | -8.94 | <0.001 |
|  | Brand (Ref.: Brand A) | 0.01 | 1.01 | 0.09 | 0.15 | 0.88 |
|  | Size (10% increase) | -0.19 | 0.83 | 0.02 | -10.94 | <0.001 |
|  | Packaging:brand | -0.37 | 0.69 | 0.14 | -2.58 | 0.01 |
|  | Adj-R2 | 0.10 | | | | |
|  | AIC | 4143.87 | | | | |
|  | BIC | 4172.12 | | | | |
| **TASTE** | ASC | -0.23 | 0.79 | 0.11 | -2.05 | 0.04 |
|  | Packaging (Ref.: Branded pack.) | -1.13 | 0.32 | 0.10 | -11.65 | <0.001 |
|  | Brand (Ref.: Brand A) | 0.27 | 1.31 | 0.08 | 3.30 | <0.001 |
|  | Size (10% increase) | -0.11 | 0.89 | 0.02 | -6.96 | <0.001 |
|  | Packaging:brand | -0.24 | 0.79 | 0.14 | -1.74 | 0.08 |
|  | Adj-R2 | 0.09 | | | | |
|  | AIC | 4198.74 | | | | |
|  | BIC | 4226.99 | | | | |
| **LESS HARMFUL** | ASC | 0.52 | 1.68 | 0.11 | 4.73 | <0.001 |
|  | Packaging (Ref.: Branded pack.) | -0.37 | 0.69 | 0.10 | -3.74 | <0.001 |
|  | Brand (Ref.: Brand A) | 0.30 | 1.34 | 0.09 | 3.22 | <0.001 |
|  | Size (10% increase) | -0.27 | 0.76 | 0.02 | -15.17 | <0.001 |
|  | Packaging:brand | -0.20 | 0.82 | 0.14 | -1.46 | 0.14 |
|  | Adj-R2 | 0.09 | | | | |
|  | AIC | 4216.80 | | | | |
|  | BIC | 4245.04 | | | | |

Try – preferences to try; Taste - better taste perceptions; Less harmful – less harmful perceptions. Packaging - the effect of the standardised packaging category (versus branded packaging) with the ‘Brand A’; Brand - the effect of the ‘Brand B’ (versus ‘Brand A’) standardised packaging; Size - the effect of 10% increase in the warning size; Packaging×Brand - the effect of standardised packaging (versus branded packaging) with the ‘Brand B; ASC – dummy variable. Adj-R2 - R-Squared value adjusted by the number of estimated coefficients; AIC -n Akaike information criterion; BIC – Bayesian information criterion.

**Supplementary Table 6.** Conditional Choice Sets Discrete Choice Experiment Results - adjusted for Smoking Status, Gender, and Age.

| **OUTCOME** | **ATTRIBUTE** | **coef** | **OR** | **se** | **z** | **p** |
| --- | --- | --- | --- | --- | --- | --- |
| **TRY** | PACKAGING (Ref.: Branded pack.) | -0.89 | 0.41 | 0.14 | -6.55 | <0.001 |
|  | BRAND (Ref.: Brand A) | -0.30 | 0.74 | 0.13 | -2.25 | 0.024 |
|  | SIZE (10% increase) | -0.15 | 0.86 | 0.03 | -5.94 | <0.001 |
|  | PACKAGING:BRAND | -0.20 | 0.82 | 0.12 | -1.66 | 0.098 |
|  | PACKAGING:MALE | 0.10 | 1.11 | 0.13 | 0.80 | 0.423 |
|  | BRAND:MALE | -0.20 | 0.82 | 0.12 | -1.64 | 0.101 |
|  | PACKAGING:WEEKLY-SMOKER | 0.02 | 1.02 | 0.15 | 0.16 | 0.874 |
|  | PACKAGING:NON-SMOKER | -0.74 | 0.48 | 0.17 | -4.23 | <0.001 |
|  | BRAND:WEEKLY-SMOKER | 0.86 | 2.36 | 0.15 | 5.91 | <0.001 |
|  | BRAND:NON-SMOKER | 1.33 | 3.78 | 0.16 | 8.10 | <0.001 |
|  | PACKAGING:AGE | 0.03 | 1.04 | 0.01 | 2.43 | 0.015 |
|  | BRAND:AGE | 0.03 | 1.03 | 0.01 | 1.99 | 0.047 |
|  | SIZE:WEEKLY-SMOKER | -0.04 | 0.96 | 0.04 | -1.03 | 0.301 |
|  | SIZE:NON-SMOKER | -0.14 | 0.87 | 0.04 | -3.29 | 0.001 |
|  | Adj-R2 | 0.20 | | | | |
|  | AIC | 2338.52 | | | | |
|  | BIC | 2417.61 | | | | |
| **TASTE** | PACKAGING (Ref.: Branded pack.) | -1.13 | 0.32 | 0.14 | -7.90 | <0.001 |
|  | BRAND (Ref.: Brand A) | -0.30 | 0.74 | 0.14 | -2.19 | 0.029 |
|  | SIZE (10% increase) | -0.12 | 0.89 | 0.03 | -4.61 | <0.001 |
|  | PACKAGING:BRAND | -0.30 | 0.74 | 0.12 | -2.42 | 0.015 |
|  | PACKAGING:MALE | 0.25 | 1.28 | 0.13 | 1.85 | 0.064 |
|  | BRAND:MALE | -0.35 | 0.71 | 0.13 | -2.76 | 0.006 |
|  | PACKAGING:WEEKLY-SMOKER | 0.01 | 1.01 | 0.16 | 0.08 | 0.940 |
|  | PACKAGING:NON-SMOKER | -0.63 | 0.53 | 0.18 | -3.54 | <0.001 |
|  | BRAND:WEEKLY-SMOKER | 1.30 | 3.68 | 0.15 | 8.65 | < 2e-16 |
|  | BRAND:NON-SMOKER | 1.60 | 4.96 | 0.16 | 9.73 | < 2e-16 |
|  | PACKAGING:AGE | 0.04 | 1.05 | 0.01 | 3.02 | 0.003 |
|  | BRAND:AGE | 0.04 | 1.04 | 0.01 | 2.77 | 0.006 |
|  | SIZE:WEEKLY-SMOKER | -0.01 | 0.99 | 0.04 | -0.26 | 0.791 |
|  | SIZE:NON-SMOKER | -0.06 | 0.94 | 0.04 | -1.58 | 0.114 |
|  | Adj-R2 | 0.22 | | | | |
|  | AIC | 2257.14 | | | | |
|  | BIC | 2336.23 | | | | |
| **LESS HARMFUL** | PACKAGING (Ref.: Branded pack.) | -0.21 | 0.81 | 0.13 | -1.59 | 0.112 |
|  | BRAND (Ref.: Brand A) | -0.06 | 0.94 | 0.13 | -0.47 | 0.637 |
|  | SIZE (10% increase) | -0.24 | 0.79 | 0.03 | -9.31 | <0.001 |
|  | PACKAGING:BRAND | -0.37 | 0.69 | 0.12 | -3.00 | 0.003 |
|  | PACKAGING:MALE | -0.25 | 0.78 | 0.12 | -2.16 | 0.031 |
|  | BRAND:MALE | -0.01 | 0.99 | 0.12 | -0.07 | 0.948 |
|  | PACKAGING:WEEKLY-SMOKER | 0.18 | 1.19 | 0.14 | 1.23 | 0.219 |
|  | PACKAGING:NON-SMOKER | 0.10 | 1.11 | 0.15 | 0.68 | 0.495 |
|  | BRAND:WEEKLY-SMOKER | 0.77 | 2.15 | 0.14 | 5.41 | <0.001 |
|  | BRAND:NON-SMOKER | 0.89 | 2.44 | 0.15 | 5.99 | <0.001 |
|  | PACKAGING:AGE | 0.05 | 1.05 | 0.01 | 3.84 | <0.001 |
|  | BRAND:AGE | 0.02 | 1.02 | 0.01 | 1.29 | 0.196 |
|  | SIZE:WEEKLY-SMOKER | 0.04 | 1.04 | 0.04 | 1.09 | 0.277 |
|  | SIZE:NON-SMOKER | -0.10 | 0.90 | 0.04 | -2.58 | 0.010 |
|  | Adj-R2 | 0.14 | | | | |
|  | AIC | 2489.85 | | | | |
|  | BIC | 2568.95 | | | | |

Try – preferences to try; Taste - better taste perceptions ; Less harmful – less harmful perceptions. Packaging - the effect of the standardised packaging category (versus branded packaging); Brand - the effect of the ‘Brand B’ (versus ‘Brand A’); Size - the effect of 10% increase in the warning size; SS – Smoking Status (daily smokers, weekly smokers versus no smokers); Gender (male versus female); age (continues variable -centred). Adj-R2 - R-Squared value adjusted by the number of estimated coefficients; AIC -n Akaike information criterion; BIC – Bayesian information criterion.

**Supplementary Table 7**. Unconditional Choice Sets Discrete Choice Experiment Results adjusted to Smoking Status, Gender, and Age

| **OUTCOME** | **ATTRIBUTE** | **coef** | **OR** | **se** | **z** | **p** |
| --- | --- | --- | --- | --- | --- | --- |
| **TRY** | ASC | 1.62 | 5.04 | 0.49 | 3.31 | 0.001 |
|  | PACKAGING (Ref.: Branded pack.) | -1.88 | 0.15 | 0.47 | -4.02 | <0.001 |
|  | BRAND (Ref.: Brand A) | -0.85 | 0.43 | 0.45 | -1.90 | 0.057 |
|  | SIZE (10% increase) | -0.21 | 0.81 | 0.03 | -7.41 | <0.001 |
|  | PACKAGING:BRAND | -0.33 | 0.72 | 0.15 | -2.13 | 0.034 |
|  | PACKAGING:MALE | 0.08 | 1.09 | 0.15 | 0.55 | 0.585 |
|  | BRAND:MALE | -0.34 | 0.72 | 0.15 | -2.29 | 0.022 |
|  | PACKAGING:WEEKLY-SMOKER | -0.08 | 0.92 | 0.17 | -0.49 | 0.626 |
|  | PACKAGING:NON-SMOKER | -0.47 | 0.62 | 0.25 | -1.87 | 0.061 |
|  | BRAND:WEEKLY-SMOKER | 1.19 | 3.29 | 0.16 | 7.29 | <0.001 |
|  | BRAND:NON-SMOKER | 0.95 | 2.60 | 0.22 | 4.38 | <0.001 |
|  | PACKAGING:AGE | 0.04 | 1.04 | 0.02 | 2.17 | 0.030 |
|  | BRAND:AGE | 0.02 | 1.02 | 0.02 | 0.97 | 0.333 |
|  | SIZE:WEEKLY-SMOKER | -0.05 | 0.96 | 0.04 | -1.12 | 0.265 |
|  | SIZE:NON-SMOKER | 0.08 | 1.08 | 0.05 | 1.44 | 0.149 |
|  | ASC:MALE | 0.56 | 1.76 | 0.16 | 3.56 | <0.001 |
|  | ASC:WEEKLY-SMOKER | -0.69 | 0.50 | 0.25 | -2.79 | 0.005 |
|  | ASC:NON-SMOKER | -3.59 | 0.03 | 0.35 | -10.18 | <0.001 |
|  | ASC:AGE | -0.03 | 0.97 | 0.02 | -1.68 | 0.093 |
|  | Adj-R2 | 0.23 | | | | |
|  | AIC | 3551.97 | | | | |
|  | BIC | 3659.31 | | | | |
| **TASTE** | ASC | 1.39 | 4.03 | 0.48 | 2.88 | 0.004 |
|  | PACKAGING (Ref.: Branded pack.) | -1.61 | 0.20 | 0.43 | -3.74 | <0.001 |
|  | BRAND (Ref.: Brand A) | -0.82 | 0.44 | 0.41 | -2.01 | 0.044 |
|  | SIZE (10% increase) | -0.08 | 0.92 | 0.03 | -3.21 | 0.001 |
|  | PACKAGING:BRAND | -0.12 | 0.89 | 0.14 | -0.84 | 0.402 |
|  | PACKAGING:MALE | 0.18 | 1.19 | 0.14 | 1.23 | 0.221 |
|  | BRAND:MALE | -0.43 | 0.65 | 0.14 | -3.18 | 0.001 |
|  | PACKAGING:WEEKLY-SMOKER | -0.16 | 0.85 | 0.16 | -1.00 | 0.318 |
|  | PACKAGING:NON-SMOKER | -0.92 | 0.40 | 0.21 | -4.35 | <0.001 |
|  | BRAND:WEEKLY-SMOKER | 1.23 | 3.43 | 0.16 | 7.88 | <0.001 |
|  | BRAND:NON-SMOKER | 1.21 | 3.34 | 0.18 | 6.59 | <0.001 |
|  | PACKAGING:AGE | 0.02 | 1.02 | 0.02 | 1.26 | 0.209 |
|  | BRAND:AGE | 0.02 | 1.02 | 0.02 | 1.50 | 0.134 |
|  | SIZE:WEEKLY-SMOKER | -0.06 | 0.94 | 0.04 | -1.63 | 0.103 |
|  | SIZE:NON-SMOKER | -0.07 | 0.94 | 0.04 | -1.49 | 0.135 |
|  | ASC:MALE | 0.45 | 1.57 | 0.16 | 2.86 | 0.004 |
|  | ASC:WEEKLY-SMOKER | -0.46 | 0.63 | 0.25 | -1.82 | 0.068 |
|  | ASC:NON-SMOKER | -2.54 | 0.08 | 0.30 | -8.40 | < 2e-16 |
|  | ASC:AGE | -0.04 | 0.96 | 0.02 | -2.36 | 0.019 |
|  | Adj-R2 | 0.17 | | | | |
|  | AIC | 3832.90 | | | | |
|  | BIC | 3940.24 | | | | |
| **LESS HARMFUL** | ASC | 1.55 | 4.72 | 0.43 | 3.61 | <0.001 |
|  | PACKAGING (Ref.: Branded pack.) | -1.58 | 0.21 | 0.42 | -3.76 | <0.001 |
|  | BRAND (Ref.: Brand A) | -0.69 | 0.50 | 0.42 | -1.65 | 0.099 |
|  | SIZE (10% increase) | -0.23 | 0.80 | 0.03 | -8.00 | <0.001 |
|  | PACKAGING:BRAND | -0.23 | 0.79 | 0.14 | -1.66 | 0.098 |
|  | PACKAGING:MALE | -0.06 | 0.94 | 0.14 | -0.44 | 0.658 |
|  | BRAND:MALE | 0.00 | 1.00 | 0.14 | -0.01 | 0.994 |
|  | PACKAGING:WEEKLY-SMOKER | 0.17 | 1.19 | 0.16 | 1.04 | 0.298 |
|  | PACKAGING:NON-SMOKER | 0.26 | 1.29 | 0.17 | 1.47 | 0.142 |
|  | BRAND:WEEKLY-SMOKER | 0.79 | 2.21 | 0.16 | 4.84 | <0.001 |
|  | BRAND:NON-SMOKER | 0.74 | 2.10 | 0.17 | 4.28 | <0.001 |
|  | PACKAGING:AGE | 0.05 | 1.05 | 0.02 | 2.87 | 0.004 |
|  | BRAND:AGE | 0.02 | 1.02 | 0.02 | 1.36 | 0.175 |
|  | SIZE:WEEKLY-SMOKER | -0.06 | 0.94 | 0.04 | -1.45 | 0.148 |
|  | SIZE:NON-SMOKER | -0.10 | 0.91 | 0.05 | -2.17 | 0.030 |
|  | ASC:MALE | 0.54 | 1.72 | 0.14 | 3.89 | <0.001 |
|  | ASC:WEEKLY-SMOKER | -0.17 | 0.84 | 0.24 | -0.69 | 0.490 |
|  | ASC:NON-SMOKER | -0.31 | 0.73 | 0.26 | -1.22 | 0.224 |
|  | ASC:AGE | -0.05 | 0.95 | 0.02 | -2.87 | 0.004 |
|  | Adj-R2 | 0.11 | | | | |
|  | AIC | 4091.68 | | | | |
|  | BIC | 4199.02 | | | | |

Try – preferences to try; Taste - better taste perceptions; Less harmful – less harmful perceptions. Packaging - the effect of the standardised packaging category (versus branded packaging); Brand - the effect of the ‘Brand B’ (versus ‘Brand A’); Size - the effect of 10% increase in the warning size; ASC – dummy variable; SS – Smoking Status (daily smokers, weekly smokers versus no smokers); Gender (male versus female); age (continues variable -centred). Adj-R2 - R-Squared value adjusted by the number of estimated coefficients; AIC -n Akaike information criterion; BIC – Bayesian information criterion.


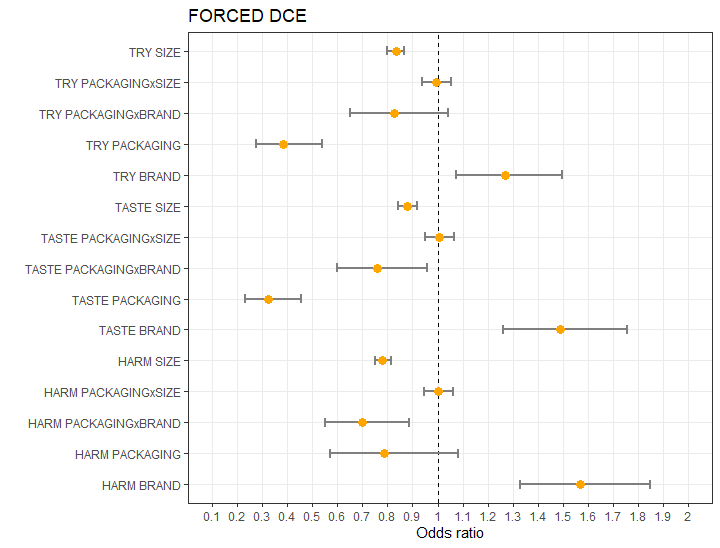

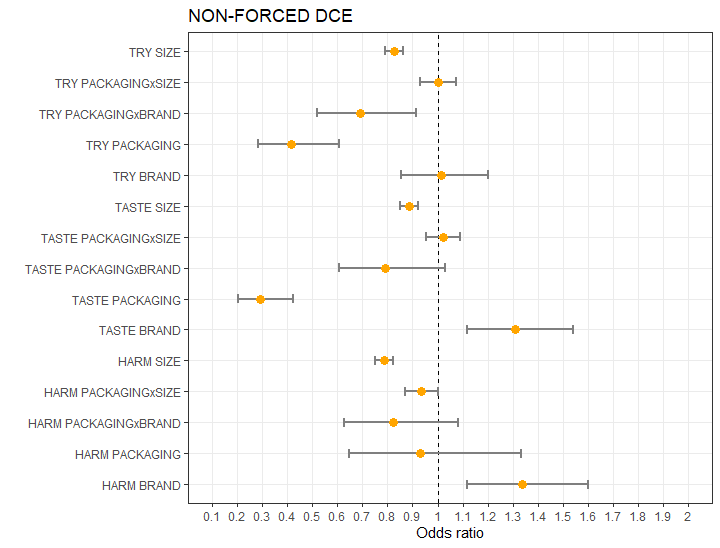


**Conditional trials**

**Unconditional trials**

**Supplementary Figure 1**. Conditional and Unconditional Results of Suboptimal Models.


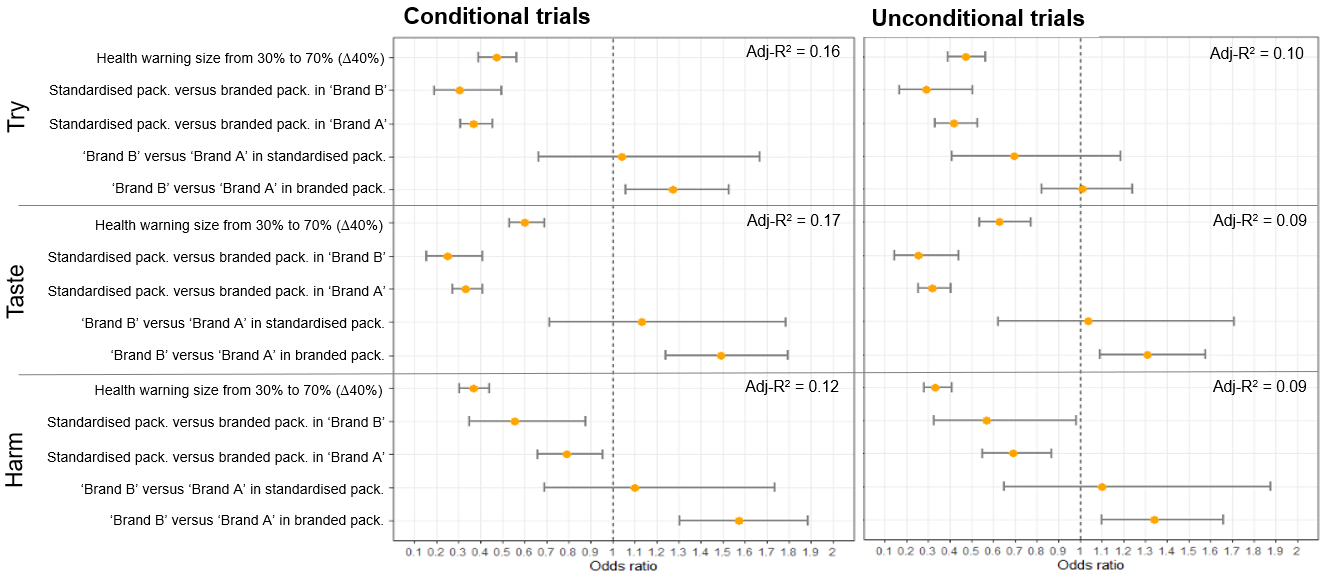
 **Supplementary Figure 2. Conditional and Unconditional Results Models with Bonferroni Adjustment**.

**Supplementary Table 8**. Suboptimal model - Conditional Choice Sets Discrete Choice Experiment Results – unadjusted.

| **OUTCOME** | **ATTRIBUTE** | **coef** | **OR** | **se** | **z** | **p** |
| --- | --- | --- | --- | --- | --- | --- |
| **TRY** | PACKAGING | -0.95 | 0.39 | 0.17 | -5.61 | <0.001 |
|  | BRAND | 0.24 | 1.27 | 0.08 | 2.80 | 0.01 |
|  | SIZE | -0.18 | 0.83 | 0.02 | -8.63 | < 2e-16 |
|  | PACKAGING:BRAND | -0.19 | 0.82 | 0.12 | -1.61 | 0.11 |
|  | PACKAGING:SIZE | -0.01 | 0.99 | 0.03 | -0.19 | 0.85 |
|  | Adj-R2 | 0.16 | | | | |
|  | AIC | 2435.17 | | | | |
|  | BIC | 2463.42 | | | | |
| **TASTE** | PACKAGING | -1.12 | 0.32 | 0.17 | -6.60 | <0.001 |
|  | BRAND | 0.40 | 1.49 | 0.08 | 4.69 | <0.001 |
|  | SIZE | -0.01 | 0.99 | 0.00 | -6.03 | <0.001 |
|  | PACKAGING:BRAND | -0.28 | 0.76 | 0.12 | -2.32 | 0.02 |
|  | PACKAGING:SIZE | 0.00 | 1.00 | 0.00 | 0.20 | 0.84 |
|  | Adj-R2 | 0.17 | | | | |
|  | AIC | 2407.18 | | | | |
|  | BIC | 2435.43 | | | | |
| **LESS HARMFUL** | PACKAGING | -0.24 | 0.78 | 0.16 | -1.48 | 0.14 |
|  | BRAND | 0.45 | 1.57 | 0.08 | 5.32 | <0.001 |
|  | SIZE | -0.25 | 0.78 | 0.02 | -11.74 | < 2e-16 |
|  | PACKAGING:BRAND | -0.36 | 0.70 | 0.12 | -2.95 | <0.001 |
|  | PACKAGING:SIZE | 0.00 | 1.00 | 0.03 | 0.06 | 0.95 |
|  | Adj-R2 | 0.12 | | | | |
|  | AIC | 2552.77 | | | | |
|  | BIC | 2581.02 | | | | |

Try – preferences to try; Taste - better taste perceptions; Less harmful – less harmful perceptions. Packaging - the effect of the standardised packaging category (versus branded packaging); Brand - the effect of the ‘Brand B’ (versus ‘Brand A’); Size - the effect of 10% increase in the warning size. Adj-R2 - R-Squared value adjusted by the number of estimated coefficients; AIC -n Akaike information criterion; BIC – Bayesian information criterion.**Supplementary Table 9**. Suboptimal model – Unconditional Choice Sets Discrete Choice Experiment Results – unadjusted.

| OUTCOME | **ATTRIBUTE** | **coef** | **OR** | **se** | **z** | **p** |
| --- | --- | --- | --- | --- | --- | --- |
| TRY | ASC | -0.01 | 0.99 | 0.16 | -0.09 | 0.93 |
|  | PACKAGING | -0.88 | 0.42 | 0.19 | -4.52 | <0.001 |
|  | BRAND | 0.01 | 1.01 | 0.09 | 0.15 | 0.88 |
|  | SIZE | -0.19 | 0.83 | 0.02 | -8.85 | < 2e-16 |
|  | PACKAGING:BRAND | -0.37 | 0.69 | 0.14 | -2.58 | 0.01 |
|  | PACKAGING:SIZE | 0.00 | 1.00 | 0.04 | 0.02 | 0.99 |
|  | Adj-R2 | 0.10 | | | | |
|  | AIC | 4145.87 | | | | |
|  | BIC | 4179.77 | | | | |
| TASTE | ASC | -0.29 | 0.75 | 0.15 | -1.91 | 0.06 |
|  | PACKAGING | -1.23 | 0.29 | 0.19 | -6.49 | <0.001 |
|  | BRAND | 0.27 | 1.31 | 0.08 | 3.30 | <0.001 |
|  | SIZE | -0.12 | 0.89 | 0.02 | -5.92 | <0.001 |
|  | PACKAGING:BRAND | -0.24 | 0.79 | 0.14 | -1.74 | 0.08 |
|  | PACKAGING:SIZE | 0.02 | 1.02 | 0.03 | 0.59 | 0.56 |
|  | Adj-R2 | 0.09 | | | | |
|  | AIC | 4200.40 | | | | |
|  | BIC | 4234.30 | | | | |
| LESS HARMFUL | ASC | 0.68 | 1.98 | 0.14 | 4.88 | <0.001 |
|  | PACKAGING | -0.07 | 0.93 | 0.18 | -0.40 | 0.69 |
|  | BRAND | 0.29 | 1.34 | 0.09 | 3.19 | <0.001 |
|  | SIZE | -0.24 | 0.79 | 0.02 | -10.41 | < 2e-16 |
|  | PACKAGING:BRAND | -0.20 | 0.82 | 0.14 | -1.41 | 0.16 |
|  | PACKAGING:SIZE | -0.07 | 0.93 | 0.04 | -1.90 | 0.06 |
|  | Adj-R2 | 0.09 | | | | |
|  | AIC | 4215.16 | | | | |
|  | BIC | 4249.05 | | | | |

Try – preferences to try; Taste - better taste perceptions; Less harmful – less harmful perceptions. Packaging - the effect of the standardised packaging category (versus branded packaging); Brand - the effect of the ‘Brand B’ (versus ‘Brand A’); Size - the effect of 10% increase in the warning size; ASC – dummy variable. Adj-R2 - R-Squared value adjusted by the number of estimated coefficients; AIC -n Akaike information criterion; BIC – Bayesian information criterion.
